# NDRG1 in Cancer: A Suppressor, Promoter, or Both?

**DOI:** 10.3390/cancers14235739

**Published:** 2022-11-22

**Authors:** Vaibhavi Joshi, Sunil R. Lakhani, Amy E. McCart Reed

**Affiliations:** 1 UQ Centre for Clinical Research, Faculty of Medicine, The University of Queensland, Brisbane 4029, Australia; 2 Pathology Queensland, The Royal Brisbane and Women’s Hospital, Brisbane 4029, Australia

**Keywords:** NDRG1, metastasis, tumour suppressor, therapeutic target, biomarker

## Abstract

**Simple Summary:**

NDRG1 (N-myc downregulated gene-1) has been reported to suppress metastasis, to be a biomarker of poor outcome, and to be a facilitator of disease progression in a range of different cancers. Characterizing NDRG1 remains challenging due to its pleiotropic functions. This review summarizes the role of NDRG1 in cancer and provides an overview of its expression and function in different cancer types.

**Abstract:**

N-myc downregulated gene-1 (NDRG1) has been variably reported as a metastasis suppressor, a biomarker of poor outcome, and a facilitator of disease progression in a range of different cancers. NDRG1 is poorly understood in cancer due to its context-dependent and pleiotropic functions. Within breast cancer, NDRG1 is reported to be either a facilitator of, or an inhibitor of tumour progression and metastasis. The wide array of roles played by NDRG1 are dependent on post-translational modifications and subcellular localization, as well as the cellular context, for example, cancer type. We present an update on NDRG1, and its association with hallmarks of cancer such as hypoxia, its interaction with oncogenic proteins such as p53 as well its role in oncogenic and metastasis pathways in breast and other cancers. We further comment on its functional implications as a metastasis suppressor and promoter, its clinical relevance, and discuss its therapeutic targetability in different cancers.

## 1. Introduction

N-myc downregulated gene-1 (NDRG1) is often called a metastasis suppressor protein and is found to be expressed in various tumour types. It is an intracellular protein composed of 394 amino acids and is 43 kD in size with several isoforms reported [1]. NDRG1 is involved in cellular processes such as stress responses, immunity, and DNA repair. In recent years, interest in the role of NDRG1 in cancer progression has increased and it is classified as both a pro-tumorigenic and tumour-suppressive protein [2]. Herein, we will broadly consider the function of NDRG1, as well as its role in different cancer types and metastasis.

## 2. NDRG1: History and Structure

*NDRG1* gene homologs are highly conserved across species (plants, humans, *C. elegans*, and mice [3]), indicating its importance in cellular function. Initial studies showed NDRG1 to be associated with cell differentiation in normal and tumour tissues. NDRG1 expression was first found to be upregulated upon cell differentiation in colon epithelial cells and downregulated in colon cancer [4]. Originally referred to as *NDR1*, *NDRG1* was reported to be a target gene repressed by oncoproteins N-myc and c-myc, with upregulation in N-myc mutant mouse embryos [5]. As high-risk cancers often abnormally express myc [6], myc-mediated regulation of NDRG1 makes this protein an interesting therapeutic candidate in cancer.

**Figure 1:**
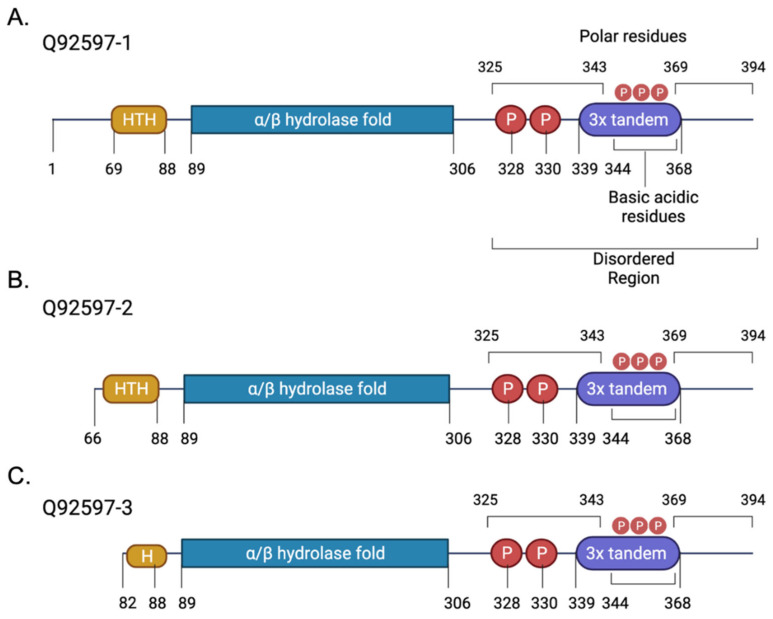
Alternatively spliced NDRG1 isoforms. (**A**) Canonical NDRG1 sequence (full length NDRG1). (**B**) Isoform 2 with 66 amino acid (1–66) difference from the canonical sequence. (**C**) Isoform 3, with 81 amino acid (1–81) difference from the canonical sequence [Made with Biorender; modified from [1]].

The *NDRG1* gene maps to chromosome 8q24.3 and encodes a 3.0 kb mRNA, which further translates to a 43 kD protein. Three alternatively spliced isoforms of NDRG1 have been reported: Q92597-1 (43 kD, Figure 1A); Q92597-2 (35 kD, Figure 1B); and, Q92597-3 (33 kD, Figure 1C) [7]. The NDRG1 promoter region consists of repeated CpG islands [8] regions, which are often observed as sites of DNA methylation mediated gene regulation in cancers and tumour suppressor genes [9]. NDRG1 shares a 53–65% sequence identity with other NDRG proteins (NDRG2, NDRG3, and NDRG4); the greatest identity between these forms of NDRG lies at the α/β hydrolase motif. Intriguingly, none of the NDRG isoforms exhibit hydrolase activity [10] but this domain allows the proteins to undergo extensive post-translational modification [11]. NDRG1 is structurally different from other NDRG proteins due to the presence of three tandem repeats of 10 amino acids (GTRSRSHTSE) in the C terminus [12], and a helix-turn-helix (HTH) at the N-terminus of the protein sequence [13]; the roles of these features are not yet evident. NDRG1 contains multiple phosphorylation (P) sites [14] and its phosphorylation is mediated via glycogen synthase kinase 3 (GSK3), a central protein in the Wnt signalling pathway. In fact, NDRG1 phosphorylation at Ser330 and THr346 was shown to suppress the NF-κB signalling pathway in pancreatic cancer cells [15].

## 3. NDRG1 Expression and Localization

**Figure 2:**
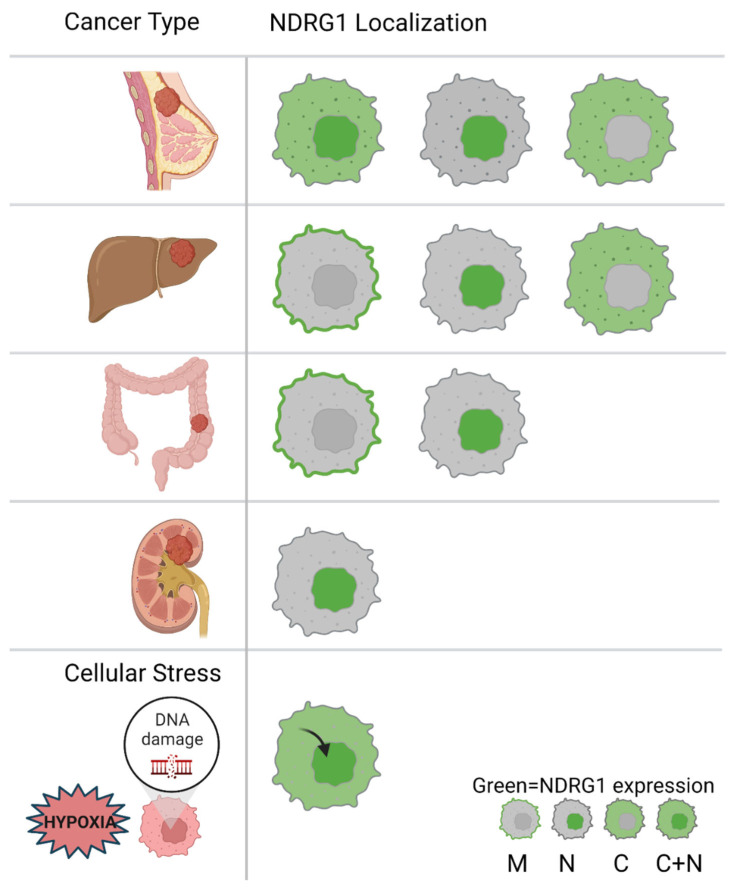
Subcellular localization of NDRG1 in different types of cancer & under cellular stress. C, Cytoplasm; M, Membrane; N, Nucleus.

NDRG1 was reported to be mostly expressed in epithelial cells with sparse presentation in mesenchymal or endothelial cells [16]. Subcellularly, NDRG1 exhibits a diverse pattern of localization and is seen to be dominantly cytoplasmic, with some presence in the plasma membrane and nucleus [16]. NDRG1 sub-cellular localization varies according to the tissue type (Figure 2). For example, NDRG1 co-localizes with the plasma membrane in intestinal and lactating breast epithelial cells, whereas nuclear localization was noted for prostate epithelial cells, and dominant cytoplasmic expression in the kidney [16]. NDRG1 localization is also responsive to exogenous stimuli. For example, in DNA damage contexts, NDRG1 was shown to translocate to the nucleus in bladder carcinoma cells [17], while hypoxic conditions in trophoblasts enhanced NDRG1 expression in the nucleus and cytoplasm [13]. The translocation of NDRG1 under hypoxic and DNA damaging conditions supports its function as a stress responsive gene [18]. Additionally, both phosphorylation and proteolytic cleavage of NDRG1, shown in HCC, prostate, colon, and pancreatic cancer cells have been associated with different localization implications [1]. The non-cleaved NDRG1 along with Ser300 p-NDRG1 were shown to be cytosolic whereas the cleaved form of NDRG1 along with Thr346 p-NDRG1 were shown to localize in the nucleus.

NDRG1 was observed to co-localize with specific cellular organelles [16] and in some cases linked to specific cellular functions. For example, in prostate cancer cells NDRG1 was found to colocalize with markers for recycling endosomes, as well as for late endosomes [19], and was shown to be involved in the recycling of E-cadherin. NDRG1 was also found to be expressed in the mitochondrial inner membrane in the proximal tubule cells of the kidney [16] and was later found to weakly bind to cardiolipin, a mitochondrial inner membrane protein, in prostate cancer cells [19], indicating a possible role in mitochondrial apoptotic processes [20]. Under hypoxic conditions, NDRG1 localized to desmosomes, endoplasmic reticulum, and perinuclear tubulin in trophoblasts, but was not observed in trophoblastic mitochondria or trans golgi network [13]. NDRG1 was also shown to localize to the microtubule matrix of the centrosome, where it protects against spindle disruption [21]. Interestingly, NDRG1 overexpression in p53-deficient tumour cell lines resulted in rescuing the cells from a spindle damage induced-mitosis arrest, indicating NDRG’s involvement in regulation of microtubule dynamics and cell cycle. Phosphorylated NDRG1 was shown to colocalize with the γ-tubulin on centromeres and at the cleavage furrow during cytokinesis further supporting a role in controlling microtubule function [22]. 

## 4. Hypoxia and NDRG1

NDRG1 is a stress responsive protein, and its association with hypoxia, a major pathological stress process associated with cancer progression [23], has been investigated in several studies [24,25,26]. The non-coding sequence of the *NDRG1* gene consists of three hypoxia inducible factor one (HIF-1) binding sites, one in the promoter and two in the 3′ untranslated region. HIF-1 binding sites in the 3′ region of the erythropoietin gene regulate its transcription [27,28], thus suggesting that *NDRG1* may similarly be regulated via HIF-1 [24]. Wang et al., later defined the minimal essential HIF-1 binding site in the NDRG1 promoter region (−1202 to −450) in lung cancer cells [29].

Under hypoxic conditions, HIF-1α was essential for *NDRG1* transcription in Hepatocellular carcinoma (HCC) cells [26,30]. Indeed, NDRG1 expression is significantly upregulated in HCCs, making it a potential indicator of malignant progression and poor prognosis [31]. Hypoxia-induced cytoplasmic localization of NDRG1 was also observed in HCC cells [30]. Furthermore, hypoxia-induced NDRG1 contributes to doxorubicin resistance in HCC cells by inhibiting apoptosis [26]. Additionally, Guo et al. [26] observed that under hypoxic conditions, NDRG1-knockdown cells showed an elevation in the accumulation of pro-apoptotic protein BAX and a decrease in anti-apoptotic proteins Bcl-2 and Bclxl, indicating NDRG1-mediated inhibition of apoptosis. This survival promotion necessitates modulation of mitochondrial dynamics, an intriguing prospect for future study.

## 5. NDRG1 and Cancer

The specific functions of NDRG1 have been examined in a number of cancer types. Here, we discuss the most well-researched of these:

### 5.1. Breast Cancer

NDRG1 expression was investigated in breast cancer (BCa) samples and was reported to be an independent prognostic factor [8,32]. Clinical analysis showed a significant reduction in NDRG1 expression in BCa patients with bone and lymph node metastasis, indicating its potential involvement in BCa progression [8]. Confusingly, *NDRG1* is located on 8q24.3, in the vicinity of *MYC*, and amplification of this region is common and is prognostic in BCa [33,34] and HCC [35].

A novel approach to treating cancer involves the use of molecules that are able to induce differentiation in highly plastic cancer cells ([36] and Section 7 and Section 8 below). The functional indication of differentiated or lineage committed cells is expression of a measurable phenotype, e.g., breast cells identified by their ability to produce milk [37]. Bae et al., established a close link between NDRG1 expression and milk-producing differentiation status in BCa cells. This study reported that drug-induced differentiation in BCa cells also resulted in an elevation in NDRG1 expression, identifying NDRG1 as a successful marker of this therapeutic approach in BCa. Though these studies established NDRG1 to be a promising target for regulating BCa progression, its role in breast cancer remains controversial due to it also being involved in metastasis progression [38,39,40]. NDRG1 was reported to contribute to BCa aggressiveness by modulating lipid metabolism [41]. Enhanced lipid synthesis and lipid uptake contributes to cancer progression and metastasis [42]. Alteration in lipid metabolism is directly linked to increased dependency on glycolysis in cancer, a hallmark of cancer progression. Luo et al., showed high NDRG1 expression to be associated with an elevation in aggressive metabolic gene signature, with a high probability of disease recurrence and metastasis. Similarly, NDRG1 silencing in HCC was shown to disrupt hypoxia-enhanced aerobic glycolysis [26].

In inflammatory BCa, NDRG1 expression has been established as a negative prognostic marker [38,39]. The estrogen receptor (ER) status is a well-established prognostic and predictive biomarker in BCa and ER-negativity is typically associated with a more aggressive phenotype and shorter overall survival. Villodre et al., stratified ER-negative patients based on NDRG1 expression levels and observed a survival difference. Their study showed that ER-negative patients with high NDRG1 expression were associated with worse survival as compared to ER-negative patients with low NDRG1 expression highlighting the potential usefulness of NDRG1 as a biomarker to increase the accuracy in predicting clinical outcomes of ER-negative patients.

### 5.2. Prostate Cancer

NDRG1 is an androgen-regulated gene, making it a central protein of interest in Prostate cancer (PrCa) [43] and a target of study for many years [44,45,46]. NDRG1 has been reported to be involved in both metastasis progression as well as suppression in PrCa [45,47]. Song et al. [47] showed that subcellular localization, specifically a decrease in membrane expression of NDRG1, was associated with significantly reduced survival outcomes in PrCa patients. Additionally, high cytoplasmic and reduced membranous expression of NDRG1 also correlated with a higher Gleason score, a scale used to predict the aggressiveness of PrCa. Knocking down NDRG1 in prostate cancer cells increased Cdc42 activity [46], a protein involved in cell cycle regulation as well as in resistance to anoikis, a process often preceding metastasis [48]. Indeed, NDRG1 inhibition was seen to increase the ability of PrCa cells to survive anoikis, contributing to their metastatic potential [46]. Androgen receptor activation is associated with PrCa progression [49] and high NDRG1 expression was associated with a reduction in activation of androgen receptor in PrCa cells via both androgen-dependent and independent signalling pathways [50]. These results indicate that high NDRG1 expression is associated with anti-tumour function in PrCa progression, making it a promising therapeutic target.

### 5.3. Pancreatic Cancer

Low NDRG1 expression has been associated with poor prognosis in pancreatic ductal adenocarcinoma [51]. Muruyama et al., showed that NDRG1 overexpression suppresses tumour growth in a xenograft mouse model of pancreatic cancer (PC) via modulation of angiogenesis [51]. Interestingly, NDRG1 decreased the expression of CXC chemokines that are involved in pro-metastasis pathways [15]. GLI1 (human glioma-associated oncogene homolog 1) is a key driver of PC metastasis [52] and is directly involved in the transcription of hepatocyte growth factor (HGF), insulin-like growth factor 1 (IGF-1), etc, which in turn can induce oncogenic signaling pathways such as NF-κB, and WNT/β-catenin signaling [53]. Geleta et al., also showed that silencing NDRG1 significantly increased GLI1 levels and decreased its inhibitory phosphorylation. Conversely, overexpression of NDRG1 in PC cells resulted in inhibition of GLI1 expression resulting in the opposite effect demonstrating the pivotal role of NDRG1 in influencing the metastasis driver GLI1 in PC [54]. NDRG1 overexpression was also independently shown to impair cell growth in PC cells via apoptotic pathways [55]. The induction of apoptosis in PC cells by NDRG1 expression suggested that NDRG1 can restrict cell death evasion and act as a tumour suppressor. Conversely, in hepatocellular carcinoma, knocking down NDRG1 expression resulted in the induction of apoptosis [56], further reinforcing the context dependent role and tissue specific outcomes.

### 5.4. Osteosarcoma

Using proteomic analyses, Hue et al., found NDRG1 to be upregulated in human osteosarcoma (OS) cells indicating it’s potential as a diagnostic biomarker of OS [57]. HER4, a transmembrane glycoprotein important in metastasis [58], is a marker of both disease progression in OS patients and increased chemotherapy resistance in OS cells [59] and Wang et al., showed that HER4 and NDRG1 interaction resulted in increased cell growth and survival in OS cells [59]. Additionally, NDRG1 overexpression in HER4-knockdown cells rescued cell growth and survival, suggesting a co-operative mechanism of NDRG1 and HER4 in promoting survival and growth in OS cells. NDRG1 expression was also reported to mediate cancer stem cell differentiation in OS cells via Wnt activation [60]. Finally, inhibition of NDRG1 expression was shown to induce apoptosis [61] as well as reduce the protein expression of VEGF and matrix metalloproteinases, key modulators of angiogenesis, in OS cells [60]. These results suggest critical links between NDRG1 and the regulation of metastasis in OS.

## 6. Metastasis

**Figure 3:**
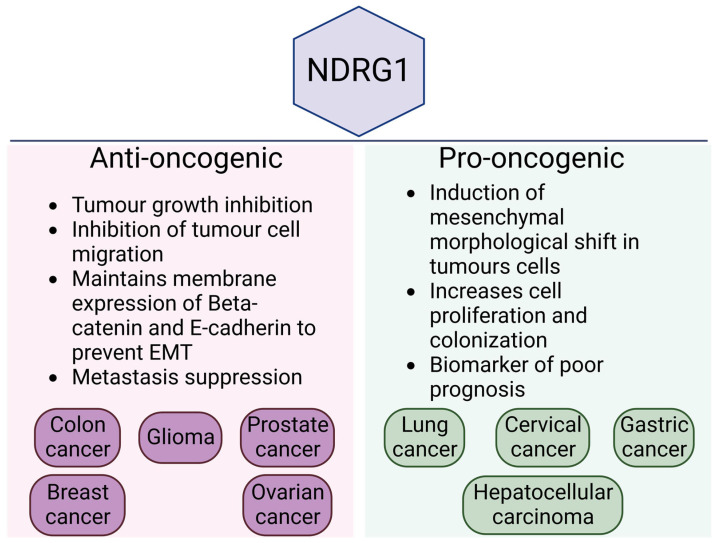
NDRG1 and its anti/pro-oncogenic roles in different cancer.

NDRG1 plays a context dependent role in cancers by either suppressing or promoting metastasis, depending on the cancer type (Figure 3). Here, we discuss NDRG1 and its link to metastasis in both contexts.

### 6.1. NDRG1: Metastasis Suppressor

The role of NDRG1 as a metastasis-suppressor protein has been widely reported in different cancers including the colon [62], prostate [45], breast [8], and pancreas [51]. Epithelial to mesenchymal transition (EMT) is a critical step in the process of metastasis [63]. Oncogenic pathways such as TGF-β and Wnt, directly and indirectly, influence metastasis progression; NDRG1 expression has been shown to be linked with these pathways. NDRG1 and Wnt signaling interplay has been investigated in the context of BCa metastasis [64]. Lie et al., found that NDRG1 interacts with LRP6 and blocks the binding of Wnt ligand to LRP6, an initiating step of the Wnt signaling cascade [64]. Upregulation of NDRG1 reduced Wnt-induced mesenchymal traits in BCa cells, along with upregulating membranous E-cadherin and β-catenin. Analysing the expression of NDRG1 and Wnt-related proteins in clinical BCa patient samples revealed a lack of NDRG1 expression in patients with Wnt protein expression, correlating with a significantly worse prognosis for metastasis free survival [64].

Cells undergoing EMT lose the expression of E-cadherin and gain a mesenchymal phenotype, allowing them to separate from the primary tumour and eventually migrate away from their original location [65]. NDRG1 was shown to inhibit EMT in colon and prostate cancer cells by modulating the transforming growth factor beta (TGF-β) pathway, a prime regulator of EMT [66]. Elevation of TGF-β signaling is commonly associated with an increase in cell invasion and metastasis in cancer [67]. TGF-β signaling activates SMAD complex which in turn activates the Snail and Slug genes resulting in elevated cell growth and survival [68]. *Snail* and *Slug* also repress the expression of the E-cadherin gene (*CDH1*) and increase the expression of cell survival related genes such as *ZEB1* and *Vimentin* (*VIM*) [69]. Guan et al. showed NDRG1 overexpression induced E-cadherin expression in SW620 colon cancer cells [62].

In prostate cancer cells, NDRG1 knockdown increased expression of SMAD complex and overexpression of NDRG1 resulted in the opposite [66] thus suggesting that NDRG1 limits the SMAD induced upregulation of *Snail*/*Slug*, and rescues the repression of E-cadherin expression. E-Cadherin expression directly enhances adhesion complex formation in cells, establishing cell–cell junctions and preventing/reducing cell motility and metastasis [66]. A unique role of NDRG1 in regulating β-catenin activity was reported by Jin et al., in colon cancer cells [66], where it was demonstrated that NDRG1 expression increases total β-catenin and non-phosphorylated β-catenin at the plasma membrane. Additionally, NDRG1 was also shown to directly block the β-catenin-nuclear translocation via inhibition of PAK4 localization. PAK4 (p21-kinase activated 4) is important in the shuttling of β-catenin to the nucleus, and hence directly affects β-catenin mediated activation of Wnt target genes [66].

The tumour suppressor PTEN (phosphatase and tensin homologue) has been shown to upregulate NDRG1 expression in breast and prostate cancers [70]. Similarly, NDRG1 was shown to upregulate PTEN expression in pancreatic as well as prostate cancer [44,71], a likely positive feedback loop. PTEN is a key tumour suppressor owing to its inhibitory role in the oncogenic PI3K pathway [44], which is critical in tumour progression and metastasis [72,73,74,75,76,77].

NDRG1 regulation was shown to be directly mediated by p53 [76] and that p53-mediated NDRG1 expression varied between metastatic lung cancer cells and non-metastatic colon cells. Furthermore, ectopic expression of p53 did not induce NDRG1 expression in H1299-p53 (metastatic lung) as compared to DLD-1-p53 (non-metastatic colon) cells. Interestingly, the study also reported that the metastatic H1299-p53 cells, which were reported to have no NDRG1 expression also lacked the expression of E-cadherin. Similarly, an absence of NDRG1 expression was reported in a metastatic colon cell line (SW620), contradicting previous ideas that NDRG1 was ubiquitously expressed across different tissues.

### 6.2. NDRG1: Pro-Metastatic Functions

Although NDRG1 function is predominantly reported as anti-oncogenic and anti-metastatic, studies also show it to be pro-oncogenic in different cancers such as gastric cancer [77] and HCC [78]. NDRG1 overexpression in clinical esophageal cancer (ESCC) samples was found to be associated with the malignant progression of the disease [79]. Indeed, NDRG1 overexpression induced a morphological change in ESCC cells, where a shift to spindle-like, mesenchymal morphology from an epithelial state was observed [80]. This was accompanied by increased expression of mesenchymal markers such as N-cadherin, Snail, and MMP1 as well as Wnt pathway-associated genes such as *WNT3A*, *LEF1*, and *FZD8*. Additionally, an increase in the accumulation of β-catenin and NDRG1 in the nuclear fraction of ESCC cells was also reported. The study hypothesized that NDRG1 impacts the Wnt pathway and β-catenin accumulation via the mediation of the Wnt-associated genes, and hence promotes metastasis [80].

NDRG1 expression is also associated with poor prognosis and malignant progression in gastric cancer [81]. NDRG1 was found to be significantly upregulated in the highly metastatic gastric cancer cell lines, as compared to the parental cells with low metastatic potential [77]. There was also an elevation in the levels of *Snail* and *VIM*, and downregulation of *CDH1* (E-cadherin). Indeed, knocking down NDRG1 in gastric cancer cells increased E-cadherin expression and suppressed the expression of vimentin; linking the typically high NDRG1 levels to the metastatic potential of gastric cancer cells. Notably, this EMT-driving interaction between NDRG1 and E-cadherin in gastric cancer is quite opposite of what was observed in the colon cancer cell line [62].

Recently, the role of NDRG1 was explored in brain metastases (BrM). Villodre et al., demonstrated that elevated levels of NDRG1 were associated with worse clinical outcomes in aggressive breast cancer [82]. Using publicly available datasets, NDRG1 expression was found to be higher in BrM than in the matched primary tumours. Additionally, NDRG1-high tumours showed reduced BrM relapse-free survival when compared to NDRG1-low tumours. This suggested a potential involvement of NDRG1 in BrM progression within the breast cancer cohort. Particularly in ER-negative patients, NDRG1 acts as a driver for BrM. Further studying NDRG1 expression in BCa xenografts and PDX tumours showed that depleting NDRG1 resulted in a decrease in migration, colony formation, invasion, and tumour initiating cells in the aggressive ER-negative BCa cells *in vitro* and *in vivo*. In 2021, it was reported that BCa-BrM development involves the slow-cycling BCa cell population which interestingly has high NDRG1 expression [83]. Depleting NDRG1 resulted in complete suppression of BrM, suggesting that NDRG1-high slow-cycling BCa cells are the dominant source of brain metastases [83].

Taken together, it is clear that NDRG1 function in tumour progression or suppression (Table 1) is highly dependent on the tumour-cell type and its differentiation status.

## 7. NDRG1 and Drug Resistance

NDRG1 expression has been associated with both chemotherapy- and radiotherapy-resistance in different cancers. Neuroblastoma cells overexpressing NDRG1 showed high resistance on treatment with clinically relevant chemotherapeutics in a multi drug treatment setting, resulting in a significantly higher cell growth relative to control cells [103]. Additionally, NDRG1 overexpression in these cells also resulted in upregulation of LRP-1, MDR, and MRP-1, proteins commonly associated with drug resistant phenotypes. Analyzing radio-resistant human rectal cancer cells revealed an elevated expression of NDRG1 at both the mRNA and protein level. Inhibition of NDRG1 in these radio-resistant cells resulted in an increase sensitization of the cell to ionizing radiation while overexpression of NDRG1 re-instilled resistance in the cells, indicating a direct role of NDRG1 in radio-resistance in rectal cancer cells [104].

Conversely, NDRG1 has also been shown to sensitize NSCLC cells to cisplatin, a common chemotherapeutic [105]. The study showed ERCC1 (excision repair cross-complementing 1) mediated NDRG1 downregulation as a key step in inducing drug resistance in the NSCLC cells. ERCC1 is a key protein for induction of cisplatin resistance and knocking it down resulted in high NDRG1 expression and a significant increase in apoptosis in NSCLC cells. Rescue experiments confirmed a strong link between ERCC1-NDRG1 modulation in drug resistance.

## 8. Therapeutic Targetability of NDRG1 in Cancer

A number of drugs have been tested for their capacity to modulate NDRG1 function. The use of novel thiosemicarbazone iron chelators as NDRG1 modulators has been explored in both cancer cells and xenograft models [106,107,108]. di-2-pyridylketone 4,4-dimethyl-3-thiosemicarbazone (Dp44mT) and di-2-pyridylketone 4-cyclohexyl-4-methyl-3-thiosemicarbazone (DpC) are iron-binding agents that have been shown to upregulate NDRG1 expression and in turn exhibit anti-metastatic activity [64,92,93]. These compounds deplete cellular iron via hypoxia-inducible factor 1α (HIF-1α) pathways [107], leading to the accumulation of HIF-1α, nuclear translocation, and formation of the HIF-1 complex [109]. The HIF-1 complex binds to hypoxia response elements (HREs) located in gene promoter regions to regulate gene expression [29,110]. Indeed, cellular iron depletion-mediated upregulation of NDRG1 expression inhibits the epithelial-mesenchymal transition that is central to the process of metastasis [66]. In the pancreatic cancer cell model, NDRG1 overexpression was induced via treatment with DpC and Dp44mT and resulted in a decrease in NF-κB activation and as well as its downstream targets Snail, Slug and, ZEB1, which suppress E-cadherin expression [111]. NDRG1 has been shown to inhibit autophagy [112], a stress responsive cellular process which is also an important regulator of tumour progression and metastasis [113]. Dp44mT mediated expression of NDRG1 in PC cells was also shown to potentiate lysosome membrane permeabilization (LMP), which inhibits autophagic degradation, leading to dysfunctional autophagy [114].

DpC and Dp44mt treatments have been shown to be a promising approach for tumour and metastasis suppression. DpC treatment in mice carrying pancreatic tumours showed a significant reduction in tumour growth and metastasis, and this was found to be more effective than the standard chemotherapy [54]. IHC analysis of these tumours revealed elevated expression of NDRG1, confirming the *in vitro* studies. Importantly DpC treatment was shown to be exhibit cancer cell specific toxicity along with significant reduction in distant metastasis [54]. In a breast cancer-bone metastasis mouse model, Dp44mt treatment over short period of time (~9 days) resulted in a dramatic decrease in circulating tumour cell burden, whereas extended treatment significantly blocked metastasis incidence with no apparent traces of drug toxicity [64]. Interestingly ablation of NDRG1 in this model resulted in resistance to treatment with Dp44mt, strongly suggesting a high selectivity between the iron chelator and NDRG1 [64]. Therapeutic efficacy iron chelators such as DpC for induction of NDRG1 expression is a significant approach with promising translational outcomes. In 2016, DpC was investigated in multi-center phase I clinical trial for treating advanced and resistant cancers (NCT02688101), making this approach more clinically relevant.

Inhibition of NDRG1 has been explored due to its pro-tumourigenic role in some cancer types. In Osteosarcoma (OS), NDRG1 was shown to play an integral role in lysosomal function. Combretastatin A-4 (CA-4) and chloroquine combination therapy has been shown to exert synergistic cytotoxic effects on human OS cells. Wang et al., showed that silencing NDRG1 sensitized OS cells to CA-4 treatment via suppression of autophagosome–lysosome fusion, potentiating an anti-tumour response [61]. NDRG1 expression was shown to be significantly increased in radio-resistant rectal cancer (RC) cell lines and its silencing was shown to sensitize RC cells to relevant clinical doses of radiation by increasing DNA double strand breaks [104]. NDRG1 suppression in both instances offers a valuable strategy to potentiate anti-tumour effects, suggesting a promising therapeutic strategy.

## 9. Conclusions/Summary

NDRG1 continues to be an intriguing mediator of cancer progression and metastasis. Its modulatory role in critical cancer signaling pathways provides evidence of its dominant influence in both tumour promotion and suppression, making it important to further explore its underlying biology. Induction of NDRG1 has been directly shown to suppress tumour growth and metastasis *in vitro* and *in vivo* in a range of different cancers. Given the diversity of NDRG1’s post translational modifications and subcellular localization, it is not surprising that it has such context dependent functions in cancer progression. Therapeutic NDRG1 induction mediated by iron-chelators such as DpC and Dp44mT is a promising intervention for cancer suppression. NDRG1 offers a wide array of opportunities for cancer treatment owing to its dynamic role.

## Figures and Tables

**Table 1:** Clinical consequences of NDRG1 expression in cancer.

Cancer Type	Oncogene or TSG?	Pre-Clinical Evidence	Clinical Relevance of NDRG1
Breast	Oncogene/TSG	↑ NDRG1, ↑ glycolytic & hypoxic genes [41] ↓ NDRG1, ↓ xenograft primary tumour growth and brain metastasis [82] Directly proportional to cell differentiation status [84] ↑ NDRG1 in slow cycling BrM initiating cells [83]	Prognostic factor for worse survival in inflammatory breast cancer [39] Significant inverse correlation DFS in other breast cancers [8]
Pancreatic	TSG	↑ NDRG1-tumour induces angiogenesis *in vivo* [51] ↑ membrane E-cadherin, supresses metastasis [85]	↑ NDRG1-Prognostic biomarker [51]
Ovarian	TSG	↓ NDRG1 promotes tumour growth *in vivo* [86] ↓ NDRG1 promotes cell adhesion and migration [87]	High NDRG1 associated with PFS * [88]
Liver	Oncogene	Promotes resistance to doxorubicin in HCC cells [25] ↓ NDRG1 tumour growth & induces senescence [89]	Indicator of poor prognosis [31] Biomarker for metastasis and recurrence [90]
Renal cell carcinoma	TSG	↓ NDRG1 enhances cell proliferation and invasion [91]	↑ NDRG1 low survival probability * [92] Prognostic biomarker for clear cell renal cell carcinoma [93]
Lung	Oncogene	↑ NDRG1 induces cell proliferation and apoptosis [29] ↑ NDRG1 induces cisplatin resistance [94]	Predictor of poor prognosis in NSCLC [95] Negatively correlated with survival and prognosis [96]
Prostate	TSG	↓ NDRG1 increases cellular invasion, directly impacting metastasis [46,97] ↓ androgen receptor signalling, ↓ EMT [50]	↓ tumour metastasis [45,46] ↓ membrane expression indicator of ↓ DFS [47]
Osteosarcoma	Oncogene/TSG	↓ NDRG1 enhances cell proliferation and invasion [98]	Biomarker for osteosarcoma [57]
Cervical cancer	Oncogene/TSG	↑ NDRG1 in invasive cervical cancer [99] ↓ tumour growth, invasion and metastasis [61]	Expression associated with poor PFS and OS [100]
Colorectal cancer	TSG	↓ EMT, migration and invasion in CRC cells [96] ↓ EGFR downstream oncogenic pathways [101]	↓ NDRG1 correlates with poor survival [102]

DFS, disease free survival; OS, overall survival; PFS, progression free survival; TSG, tumour suppressor gene; HCC, hepatocellular carcinoma. * Data mined from KM plotter and Protein atlas [88,92].
